# Supplementary material for: Comparative analysis of multiple chronic unpredictable mild stress paradigms in mice: Behavioural and physiological validity
Source: Neurobiol Stress. 2026 Apr 9;42:100815. doi: 10.1016/j.ynstr.2026.100815 (PMC13092844; doi:10.1016/j.ynstr.2026.100815)
Supplement: Multimedia component 1 [file mmc1.pdf]

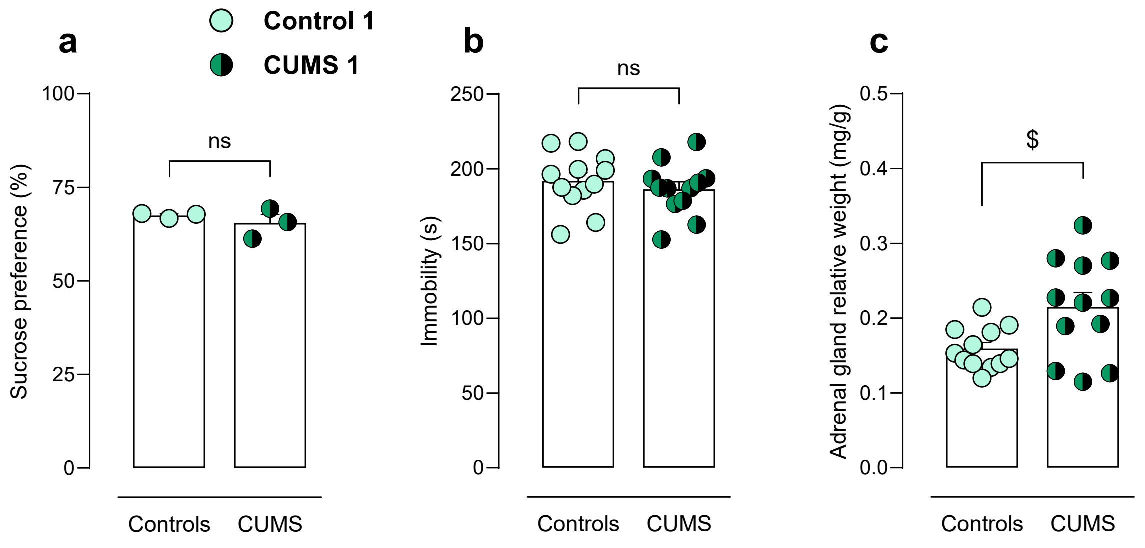

Figure S1. Behavioural and physiological evaluation of stressed and control animals in CUMS 1 protocol (n=12). **a**. Sucrose preference (%) (grouped analysis, n=3 cages/group). **b**. Immobility time in FST. **c.** Adrenal gland weight relative to bodyweight (mg/g). Unpaired *t*-test. $^{\$}p<0.05$. ns, non significant, $p>0.05$.

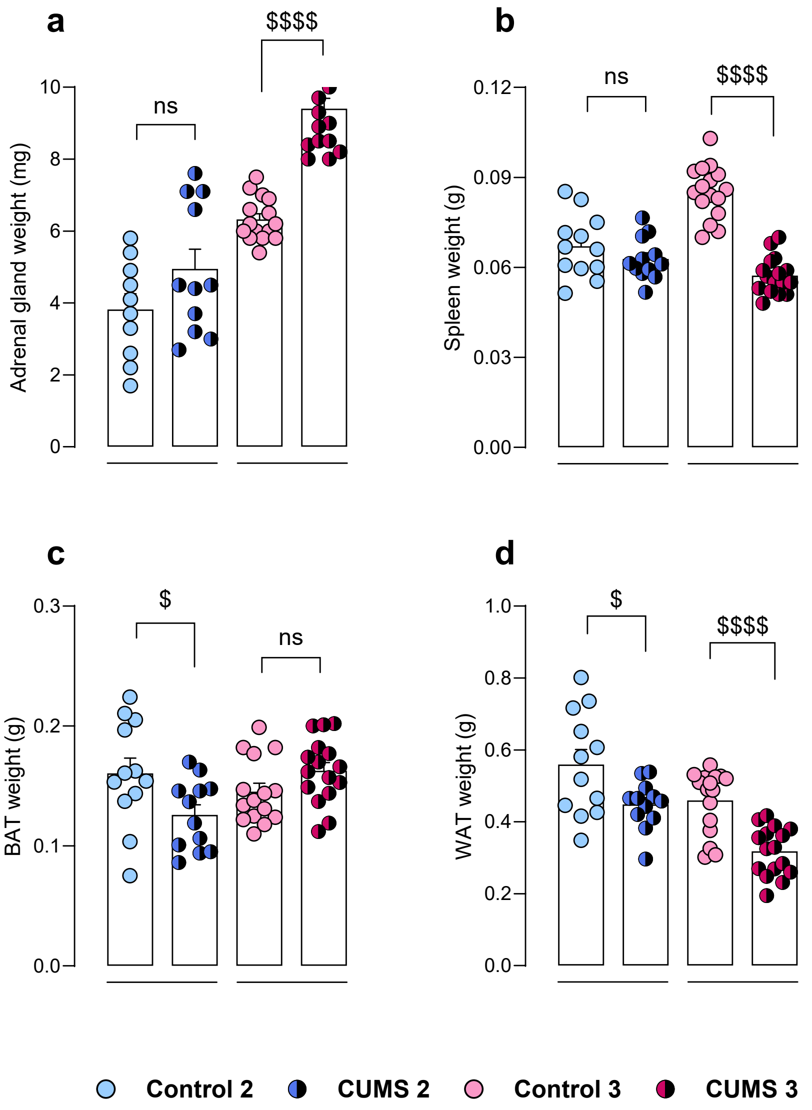

Figure S2. Absolute weight of organs in CUMS 2 (n=12) and CUMS 3 (n=16) protocols. Adrenal gland weight (**a**), spleen weight (**b**), white adipose tissue (WAT) weight (**c**) and brown adipose (BAT) tissue (**d**). Unpaired *t*-test. $^{\$}$p<0.05, $^{\$\$\$\$}$p<0.0001. ns, non significant, p>0.05.

Table S1. Stressful stimuli and description for each CUMS protocol: stressor, details, duration, phase of the cycle (D: dark, active; L: light, inactive phase) and intensity according to the severity point system. *Food/water deprivation for CUMS 3 protocol was applied for 16 hours, only before SP (food and water) and NSFT (only food) tests.

Stressor	*Description*	*Duration*	*Phase of the cycle*	*Intensity*	*1*	*2*	*3*
**Cage tilting**	Cages were tilted sideways 45º	12 h	D	1	✓	✓	✓
**Food/water deprivation**	Food and water were removed	8 h / 16 h	D	1	✓	✓	✓ *
**Space reduction**	A clear plastic wall was placed in the cage to reduce habitable space to 1/3	12 h	D	1		✓	
**Cage swap**	Sawdust from cages within the stress group was swapped	-	L	1		✓	
**Overcrowding**	8 animals were placed in an individual cage (size)	2 h	L	1			✓
**Alarm clock**	An alarm clock at 85 dB was activated at random times	10 min	L + D	2	✓	✓	✓
**White noise**	An untuned radio was set at 85 dB	4 h	L	2	✓	✓	✓
**Wet bedding**	200 mL of water were poured over 400 mL of sawdust	12 h	D	2	✓	✓	✓
**No bedding**	Sawdust was removed	12 h	D	2	✓	✓	✓
**Continuous light**	Lights were kept on for on entire sleep cycle	24 h	L + D	2	✓	✓	✓
**Light pulses**	Lights were switched on and off every 20 min	12 h	D	2		✓	✓
**Predator odour**	Sawdust was removed and replaced with sawdust from rats' cages	2 h	L	2		✓	✓
**Cold exposure**	Cages were introduced in a chamber at 4 ºC	1 h	L	3	✓	✓	✓
**Heat exposure**	A heat source was placed in the rack. Temperature did not exceed 40 ºC	2 h	L	3		✓	✓
**Stroboscopic lights**	Flashing lights were applied	4 h	L	3		✓	✓
**Water bath**	Sawdust was removed and replaced with warm (23 ºC) water (2 cm-high)	2 h	L	3		✓	✓
**Restraint (a)**	Mice were introduced in ventilated plastic cups (7 x 5 cm)	2 h	L	3		✓	
**Restraint (b)**	Mice were introduced in ventilated plastic falcons (12 x 3 cm)	2 h	L	3			✓

Table S2. Chronic stress schedule from CUMS 1 protocol.

	CUMS 1	Monday	Tuesday	Wednesday	Thursday	Friday	Saturday	Sunday
Week 1	**Stressor**	Cage tilting	Continuous light	Food/water deprivation	Cold exposure	No bedding	Alarm clock	White noise
	**Duration**	12 h	24 h	8 h	1h	12 h	10 min	4 h
Week 2	**Stressor**	Continuous light	Wet bedding	White noise	Food/water deprivation	Cage tilting	Cold exposure	Alarm clock
	**Duration**	24 h	12 h	4 h	8 h	12 h	1h	10 min
Week 3	**Stressor**	Cold exposure	No bedding	White noise	Continuous light	Wet bedding	Cage tilting	Alarm clock
	**Duration**	1h	12 h	4 h	24 h	12 h	12 h	10 min
Week 4	**Stressor**	Cage tilting	Cold exposure	Continuous light	White noise	Food/water deprivation	Wet bedding	Alarm clock
	**Duration**	12 h	1h	24 h	4 h	8 h	12 h	10 min

Table S3. Chronic stress schedule from CUMS 2 protocol.

	CUMS 2	Monday	Tuesday	Wednesday	Thursday	Friday	Saturday	Sunday
Week 1	**Stressor**	White noise	Cold exposure	Alarm clock	Cold exposure	Heat exposure	Alarm clock	Stroboscopic lights
	**Duration**	4 h	1h	10 min	1h	2 h	10 min	4 h
	**Stressor**	Stroboscopic lights	Predator odour	Restraint	Cage swap	Water bath	Cage swap	Alarm clock
	**Duration**	4 h	2 h	2 h	-	2 h	-	10 min
	**Stressor**	Food/water deprivation	Wet bedding	Continuous light	Cage tilting	Light pulses	No bedding	Space reduction
	**Duration**	8 h	12 h	24 h	12 h	12 h	12 h	12 h
Week 2	**Stressor**	Restraint	Predator odour	White noise	Stroboscopic lights	Water bath	Alarm clock	Stroboscopic lights
	**Duration**	2 h	2 h	4 h	4 h	2 h	10 min	4 h
	**Stressor**	White noise	Water bath	Cage swap	Cold exposure	Restraint	Heat exposure	Alarm clock
	**Duration**	4 h	2 h	-	1h	2 h	2 h	10 min
	**Stressor**	Wet bedding	Cage tilting	Light pulses	Continuous light	Cage tilting	Space reduction	No bedding
	**Duration**	12 h	12 h	12 h	24 h	12 h	12 h	12 h
Week 3	**Stressor**	Heat exposure	Cage swap	Cold exposure	Heat exposure	Restraint	White noise	Alarm clock
	**Duration**	2 h	-	1h	2 h	2 h	4 h	10 min
	**Stressor**	Water bath	Restraint	White noise	Stroboscopic lights	Cage swap	Predator odour	Stroboscopic lights
	**Duration**	2 h	2 h	4 h	4 h	-	2 h	4 h
	**Stressor**	Light pulses	Continuous light	Food/water deprivation	Cage tilting	Wet bedding	No bedding	Space reduction
	**Duration**	12 h	24 h	8 h	12 h	12 h	12 h	12 h
Week 4	**Stressor**	Restraint	White noise	Stroboscopic lights	Water bath	Predator odour	Alarm clock	Cage swap
	**Duration**	2 h	4 h	4 h	2 h	2 h	10 min	-
	**Stressor**	Cold exposure	Heat exposure	Cage swap	Restraint	Cold exposure	White noise	Alarm clock
	**Duration**	1h	2 h	-	2 h	1h	4 h	10 min
	**Stressor**	Wet bedding	Space reduction	Cage tilting	Continuous light	Light pulses	No bedding	Food/water deprivation
	**Duration**	12 h	12 h	12 h	24 h	12 h	12 h	8 h

Table S4. Chronic stress schedule from CUMS 3 protocol.

	CUMS 3	Monday	Tuesday	Wednesday	Thursday	Friday	Saturday	Sunday
Week 1	Stressor	Water bath	Restraint	Cold exposure	Heat exposure	Restraint	Alarm clock	Alarm clock
	Duration	2 h	2 h	1h	2 h	2 h	10 min	10 min
	Stressor	Overcrowding	Water bath	Stroboscopic lights	Water bath	Cold exposure	Alarm clock	Alarm clock
	Duration	2 h	2 h	4 h	2 h	1h	10 min	10 min
	Stressor	Predator odour		Overcrowding	White noise		Alarm clock	Alarm clock
	Duration	2 h		2 h	4 h		10 min	10 min
	Stressor	Wet bedding	Continuous light	Cage tilting	Light pulses	Wet bedding	No bedding	Continuous light
	Duration	12 h	24 h	12 h	12 h	12 h	12 h	24 h
Week 2	Stressor	Restraint	White noise	Heat exposure	Cold exposure	Water bath	Alarm clock	Alarm clock
	Duration	2 h	4 h	2 h	1h	2 h	10 min	10 min
	Stressor	Stroboscopic lights	Water bath	Restraint	Stroboscopic lights	Heat exposure	Alarm clock	Alarm clock
	Duration	4 h	2 h	2 h	4 h	2 h	10 min	10 min
	Stressor	Cold exposure	Predator odour		Overcrowding	White noise	Alarm clock	Alarm clock
	Duration	1h	2 h		2 h	4 h	10 min	10 min
	Stressor	No bedding	Wet bedding	Continuous light	Cage tilting	Light pulses	Continuous light	Continuous light
	Duration	12 h	12 h	24 h	12 h	12 h	24 h	24 h
Week 3	Stressor	Cold exposure	Stroboscopic lights	Restraint	White noise	Water bath	Alarm clock	Alarm clock
	Duration	1h	4 h	2 h	4 h	2 h	10 min	10 min
	Stressor	Water bath	Heat exposure	Stroboscopic lights	Overcrowding	Restraint	Alarm clock	Alarm clock
	Duration	2 h	2 h	4 h	2 h	2 h	10 min	10 min
	Stressor		Overcrowding		Cold exposure	White noise	Alarm clock	Alarm clock
	Duration		2 h		1h	4 h	10 min	10 min
	Stressor	No bedding	Cage tilting	Wet bedding	Light pulses	Cage tilting	Continuous light	No bedding
	Duration	12 h	12 h	12 h	12 h	12 h	24 h	12 h
Week 4	Stressor	Water bath	Restraint	Cold exposure	Heat exposure	Restraint	Alarm clock	Alarm clock
	Duration	2 h	2 h	1h	2 h	2 h	10 min	10 min
	Stressor	Overcrowding	Water bath	Stroboscopic lights	Water bath	Cold exposure	Alarm clock	Alarm clock
	Duration	2 h	2 h	4 h	2 h	1h	10 min	10 min
	Stressor	Predator odour		Overcrowding	White noise		Alarm clock	Alarm clock
	Duration	2 h		2 h	4 h		10 min	10 min
	Stressor	Wet bedding	Continuous light	Cage tilting	Light pulses	Wet bedding	No bedding	Continuous light
	Duration	12 h	24 h	12 h	12 h	12 h	12 h	24 h
Week 5	Stressor	Restraint	White noise	Heat exposure	Cold exposure	Water bath	Alarm clock	Alarm clock
	Duration	2 h	4 h	2 h	1h	2 h	10 min	10 min
	Stressor	Stroboscopic lights	Water bath	Restraint	Stroboscopic lights	Heat exposure	Alarm clock	Alarm clock
	Duration	4 h	2 h	2 h	4 h	2 h	10 min	10 min
	Stressor	Cold exposure	Predator odour		Overcrowding	White noise	Alarm clock	Alarm clock
	Duration	1h	2 h		2 h	4 h	10 min	10 min
	Stressor	No bedding	Wet bedding	Continuous light	Cage tilting	Light pulses	Continuous light	No bedding
	Duration	12 h	12 h	24 h	12 h	12 h	24 h	12 h
Week 6	Stressor	Cold exposure	Stroboscopic lights	Restraint	White noise	Water bath	Alarm clock	Alarm clock
	Duration	1h	4 h	2 h	4 h	2 h	10 min	10 min
	Stressor	Water bath	Heat exposure	Stroboscopic lights	Overcrowding	Restraint	Alarm clock	Alarm clock
	Duration	2 h	2 h	4 h	2 h	2 h	10 min	10 min
	Stressor		Overcrowding		Cold exposure	White noise	Alarm clock	Alarm clock
	Duration		2 h		1h	4 h	10 min	10 min
	Stressor	No bedding	Cage tilting	Wet bedding	Light pulses	Cage tilting	Continuous light	Food/water deprivation
	Duration	12 h	12 h	12 h	12 h	12 h	24 h	16 h

Table S5. Duration of chronic stress protocol and days of behavioural testing in CUMS 1, CUMS 2 and CUMS 3 paradigms.

		CUMS 1	CUMS 2	CUMS 3
**Behavioural test**	**Protocol duration**	28 days	28 days	42 days
Sucrose Preference test (SP)		Day 28	Day 28	Day 42
Elevated Plus Maze (EPM)		-	Day 30	Day 43
Nest-Building test (NBT)		-	Day 31	Day 44
Open Field test (OFT)		-	Day 32	Day 45
Novelty-Suppressed Feeding test (NSFT)		-	Day 33	Day 46
Tail-Suspension test (TST)		-	Day 34	Day 47
Forced Swimming test (FST)		Day 30	Day 35	Day 48
Tissue harvest		Day 32	Day 36	Day 49

Table S6. Explanation of sample sizes in each test or parameter evaluated. Outliers were detected by using the Grubbs test ($p<0.05$), and only 1 outlier was removed from each experimental group.

Parameter	Theoretical "n"	Actual "n"	Reason behind animal withdrawal
Body weight gain	56	56	-
Food intake	56	56	-
Adrenal gland relative weight	56	52	2 badly extracted tissues; 2 outliers
Spleen relative weight	56	56	-
BAT relative weight	56	55	1 outlier
WAT relative weight	56	56	-
Sucrose preference	56	49	5 leaky bottles; 2 outliers
Nest building test	56	56	-
Tail suspension test	56	43	4 mice in unrecorded video; 5 mice climbed up their tails; 4 outliers
Forced swimming test	56	55	1 outlier
Novelty suppressed feeding test	56	49	Six mice reached the maximum latency of 600 s (i.e., they did not engage in the task, displaying behaviours such as gnawing, digging, or other activities);1 outlier
Elevated plus maze	56	53	1 unrecorded video; 2 outlier
Open field test -distance-	56	55	1 outlier
Open field test –centre time-	56	53	3 outliers

Table S7. Summary of physiological and behavioural readouts in stressed groups from CUMS 1, CUMS 2 and CUMS 3 protocols. ↑, increase**.** ↓, decrease. =, no change.

	CUMS protocol		
Parameter	***1***	***2***	***3***
Bodyweight gain (%)	=	↓	↓
Food intake (g/g)		↓	↑
Adrenal gland weight (mg/g)	↑	=	↑
Spleen weight (mg/g)		=	↓
BAT weight (mg/g)		=	↑
WAT weight (mg/g)		↓	↓
Sucrose preference (%)	=	=	↓
Nest-building score		=	↓
TST immobility time (s)		=	↑
FST immobility time (s)	=	↑	↑
NSFT latency to feed (s)		=	↑
EPM time in open arms (s)		=	↓
OFT distance (cm)		↑	↑
OFT time in centre (s)		=	↓
